# Protective effect of *Glechoma hederacea* extract against gallstone formation in rodent models

**DOI:** 10.1186/s12906-021-03368-1

**Published:** 2021-07-14

**Authors:** Min Xiao, Mengbi Yang, Xiaoyu Ji, Dan Li, Yuning Xie, Yuanfeng Lyu, Zhong Zuo

**Affiliations:** School of Pharmacy, Faculty of Medicine, The Chinese University of Hong Kong, Shatin, New Territories, Hong Kong SAR, P. R. China

**Keywords:** *Glechoma hederacea* extract, Hitrechol®, Herbal medicine, Cholesterol gallstone, Lithogenic diet, Bile composition

## Abstract

**Background:**

Our current study aimed to evaluate the effect of an *Glechoma hederacea* extract (Hitrechol®) in normal rats and gallstone diseased mice to explore its underlying mechanisms. Normal rats and C57BL/6 mice with/without cholesterol gallstone were used in this study.

**Methods:**

To monitor the effect of Hitrechol® on bile secretion, bile flow rates at 15 min interval until 2 h post-dosing in normal rats treated with vehicle and Hitrechol® were compared using multiple t-test with a *p* < 0.05 considered as statistically significant different. To further evaluate the effect of Hitrechol® against the development of gallstone in lithogenic diet treated mice, mice were treated with vehicle or Hitrechol® (QD-once daily or TID-three times daily) for 3 weeks followed by comparing the levels of bile composition among the treatment groups. In addition, the anti-oxidative biomarkers in liver and anti-inflammatory biomarkers in serum were detected and compared among all the treatment groups to evaluate the hepato-protective effect of Hitrechol®. The obtained levels of biomarkers and bile composition were compared among different treatment groups using one-way ANOVA tests followed by Tukey’s multiple comparisons with *p* < 0.05 considered as statistically significant.

**Results:**

Despite no significant impact on the bile flow rate, Hitrechol® TID treatment dramatically decreased size and amount of gallstone crystals and total cholesterol level (*p < 0.05*), as well as total bile acid (*p < 0.05*) and several types of bile acid (*p < 0.05*) levels in gallstone disease model mice. Hitrechol® TID treatment could significantly decrease the frequencies of hepatocyte necrosis and lipid aggregation notably as well as increase the antioxidant enzyme level (*p < 0.05*) in the liver.

**Conclusions:**

Our findings for the first time demonstrated the beneficial effect of Hitrechol® against gallstone via its litholytic, liver-protective and antioxidant activities.

**Supplementary Information:**

The online version contains supplementary material available at 10.1186/s12906-021-03368-1.

## Background

Gallstone disease is one of the most common digestive diseases, affecting 10–20% of the global adult population [1]. More than 90 % of gallstones are cholesterol solid crystals formed in the gallbladder. The factors for causing cholesterol gallstone disease include improved standards of living, chronic high cholesterol diet, and overnutrition. In the normal physiological state, bile is produced in the liver and is secreted into the duodenum to digest food [2]. Bile contains bile salts, phospholipids, cholesterol, proteins, and bilirubin, all in stable equilibrium states [2]. However, under certain pathophysiological conditions, especially the supersaturated bile of cholesterol, the relative excess cholesterol will precipitate as solid crystals, then aggregate, fuse, and eventually form gallstones within a gallbladder-secreted mucin gel that cause disease and complaints [2, 3]. Although laparoscopic cholecystectomy is currently considered the gold standard in treating patients with symptomatic gallstones, new perspectives regarding medical therapy of cholesterol gallstone disease are under discussion. The natural history of the disease and the analysis of the overall costs of therapy are also taking into considerations [4]. Classical oral litholysis like ursodeoxycholic acid (UDCA) has been used for the treatment of cholesterol gallstone disease [4]. Recent studies have raised the possibility that cholesterol-lowering agents that inhibit hepatic cholesterol synthesis or intestinal cholesterol absorption, or drugs acting on specific nuclear receptors involved in cholesterol and bile acid homeostasis for treatment of cholesterol gallstone disease. Other natural plant extraction medicines or components are also reported to have the effect of dissolving gallstone and prevent the occurrence of this disease [5].

Hitrechol® is an herbal medicine used for gallstone disease since 1970s and was indicated for the treatment of early-stage cholesterol gallstones as well as solitary and multiple cholesterol gallstones. Hitrechol® contains the purified extract of *G. hederacea*, in which saponins, essential oil, and phenolic compounds (such as flavonoids, tannins, caffeic acid, and chlorogenic acid [6]) served as the major components. The chemical structure of the saponin ursolic acid in Hitrechol® is similar to that of UDCA, one of the compositions of bile acids in mice gallbladder reported to be effective in dissolving gallstones by lysis of the cholesterol crystals [7, 8], and in altering the bile secretion [9]. The saponins and the essential oil could significantly decrease total cholesterol level [10, 11]. Some studies have also implied that the active components of *G. hederacea* have other possible pharmacological activities including anti-inflammatory [12], antispasmodic [13], liver-protective [14], choleretic [9], litholytic [15], antioxidant [16], antimicrobial [17] and antitumor [18]. It can reduce the inflammatory process which caused by mechanical irritation attributable to gallstones in the gallbladder wall and help to relax the muscle cells in the bile ducts, facilitating the flow of bile. The plants extract can also exhibit antioxidative effect on damaged liver cells. Although several clinical studies have shown that extracts of *G.hederacea* can decrease gallbladder complaints associated with gallstone [19] and reduce the average stone area, the underlying action mechanisms of Hitrechol® remain unknown.

The current study was proposed aiming to investigate the effect of Hitrechol® against gallstone formation in developed lithogenic disease model mice and to evaluate its effect on bile flow in rats. In addition to total cholesterol and total phospholipid levels in bile, the morphological changes of gallstone crystals and pathological changes in the liver after Hitrechol® treatment were monitored. To further evaluate the liver protective effect of Hitrechol®, the antioxidant and anti-inflammatory biomarkers of catalase activities, and superoxide dismutase, reduced glutathione levels and TNF-alpha were also determined and compared among different treatment groups.

## Materials and methods

### Quality control and HPLC fingerprint of Hitrechol®

Hitrechol Aihuo Dantong (Hitrechol®) Capsule were sponsored by Pharmazeutische Fabrik Evers GmbH & Co. KG (Batch No. 25003). The major component of Hitrechol® is the extract of *G. hederacea*, which is cultivated in Central Europe, growing in shady places. It is a perennial hairy herb with creeping stem and commonly known as “ground ivy” [17]. Authentication of *G. hederacea* was conducted by expert in botany from Albrecht-von-Haller-Institute for Plant Science, the Georg-August-University Göttingen. Manufacturing of Hitrechol® by Pharmazeutische Fabrik Evers GmbH & Co. KG company, complied with all relevant regulations. Briefly, to prepare the extract, the dry herb was cut into a defined size of 1 cm followed by extraction with analytical pure alcohol (90% v/v) at room temperature (25 °C) and subsequent concentration under vacuum. The concentrated extract was then mixed with sunflower oil to form into a homogenized mixture to be encapsulated into a soft gelatin capsule.

Besides using macroscopic and microscopic identity tests, the High-Performance Liquid Chromatography (HPLC) fingerprint indicated the existence of three marker components namely ursolic acid, oleanolic acid and linolenic acid in Hitrechol® (Fig. S1). Moreover, content of ursolic acid, one of the major active marker components, was determined by HPLC with UV detector and listed in the specification of Hitrechol® (Table S1). Characteristics, identity, purity and content assay were listed in Hitrechol® specification of the batch (No. 25003) (Table S1), in which includes appearance, filling variation, disintegration time, TLC fingerprint of active ingredient, purity, microbial limit, and the contents of three active marker components. Test results of the batch comply with all the specifications with sum of ursolic acid and oleanolic acid of 40–150 μg per capsule and linolenic acid of 70–350 μg per capsule.

### Chemicals and reagents

Hitrechol Aihuo Dantong (Hitrechol®) Capsule were sponsored by Pharmazeutische Fabrik Evers GmbH & Co. KG (Batch No. 25003). Lithogenic diet was purchased from Teklad Diets EVIGO USA (#TD.88051, Cocoa Butter Diet and Purina Mouse Chow). PEG 400 was purchased from Sigma-Aldrich. Assay kit for Cholesterol/Cholesteryl Ester Quantitation Assay kit (ab65359), Phospholipid Assay Kit (ab234050), Catalase Activity Assay Kit (ab83464), Superoxide Dismutase Activity Assay kit (ab65354), GSH/GSSG Ratio Detection Assay kit (ab138881) and Mouse TNF alpha ELISA Kit (ab208348) were purchased from Abcam, UK. All other reagents were at least of analytical grades and were used without further purification.

### Evaluation of the effect of Hitrechol® on bile secretion flow rate in rats

The experiments were carried out after approval by the Animal Ethics Committee of The Chinese University of Hong Kong (Ref No. 20–007-MIS-4-B) and complied with the guidelines of the Animal Care and Utilization Committee in Hong Kong SAR. Sprague-Dawley rats were supplied and fasted overnight before experiment. Before surgery, the rats (*n* = 6/group) were anesthetized with an intraperitoneal injection of a mixed solution containing 37.5 mg/mL ketamine and 5 mg/mL xylazine at 0.2 mL per 100 g body weight. During the surgery, the body temperature of the rats was maintained at around 37 °C by a heating pad and heating lamp. The abdomen was opened by midline incision, followed by identifying the liver and common bile duct. The transition zone between the proximal bile duct and pancreas were ligated (6–0 silk). Above the distal ligature, the common bile duct was cannulated with a 6-cm polyethylene tubes (0.4 mm i.d. × 0.8 mm o.d) for bile collection. The duodenum was identified, and a flexible PVC tubing (3 mm i.d. × 5 mm o.d.) was cannulated into the beginning of duodenum. Bile flow was recorded for 30 min before treatment. Hitrechol® (suspension in saline at 0.06 g/mL, 0.12 g/kg/day, equivalent to 1.2 g/60 kg/day in human) and same volume of saline were given to rats from treatment (*n* = 6) and control groups (*n* = 6) via the PVC tubing to duodenum. Bile was collected within a 15 min interval until 2-h post-dosing. The volume of bile collected during each time interval was measured followed by calculating the bile flow and relative bile flow as follows:

$$ \mathrm{Bile}\ \mathrm{Flow}\ \left(\mathrm{mL}/\min \right)=\mathrm{bile}\ \mathrm{volume}\ \left(\mathrm{mL}\right)/\mathrm{time}\ \mathrm{interval}\ \left(\min \right) $$

$$ \mathrm{Relative}\ \mathrm{Bile}\ \mathrm{flow}\ \left(\%\right)=\frac{\mathrm{bile}\kern0.17em \mathrm{flow}\kern0.17em \mathrm{after}\kern0.17em \mathrm{dose}\ \left(\mathrm{mL}/\min \right)}{\mathrm{Mean}\ \mathrm{of}\ \mathrm{bile}\ \mathrm{flow}\ \mathrm{before}\ \mathrm{dose}\ \left(\mathrm{mL}/\mathrm{mine}\right)}\times 100\;\left(\%\right) $$

Comparisons of the Relative Bile Flow between control and Hitrechol® treatment groups were conducted using multiple t-test with a *p* < 0.05 considered as statistically significant.

### Evaluation of the effect of Hitrechol® against the development of gallstone in normal and cholesterol gallstone model mice

#### Oral administration preparations of Hitrechol®

Hitrechol® capsule content (oily form) was dispersed in aqueous solution consisting of 50% PEG400 (1:10 v/v) to serve as the preparation for oral administration.

#### Animal treatment

**Fig. 1:**
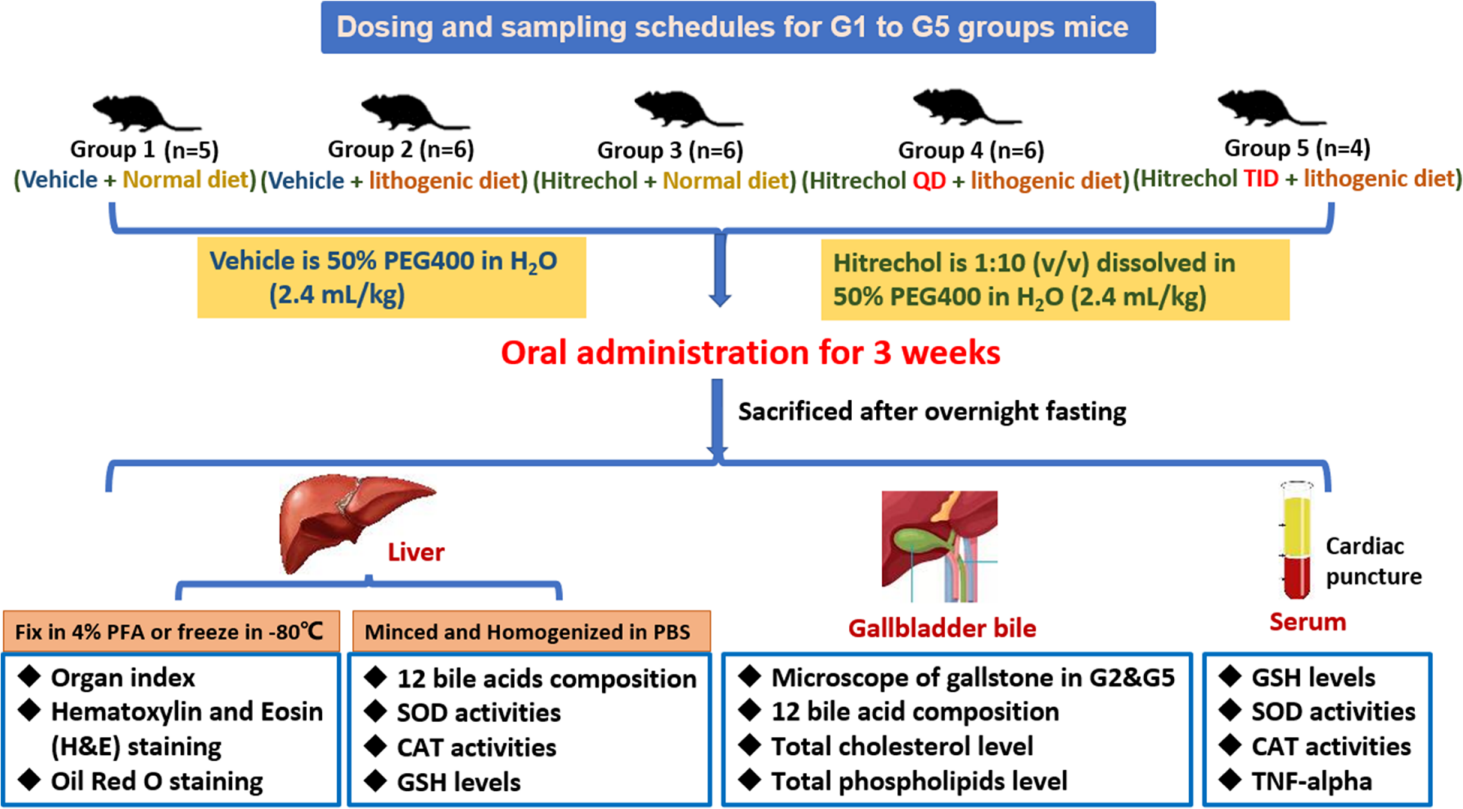
Dosing regimen and schedules of sampling and treatment for mice

The animal care and experimental procedures complied with the guidelines of the Animal Care and Utilization Committee in Hong Kong. The experiments were carried out after approval by the Animal Ethics Committee of The Chinese University of Hong Kong (Ref No. 20–007-MIS-4-B). 27 healthy male C57BL/6 mice of body weight about 22 g were kept in a controlled environment with 12/12 h light/dark cycles with free access to food and water. The mice were randomly divided into five groups (*n* = 4–6/group). Group 1 mice orally received the same volume of 50% PEG400 in H_2_O as vehicle control for 3 weeks. Group 3 mice received once daily Hitrechol® at 2.4 mL/kg (assuming the density of Hitrechol® is 1 mg/ml: equal to 2.4 g/kg in mice and 1.2 g/60 kg/day in human). Mice from Groups 2, 4 & 5 were freely access to a lithogenic diet containing approximately 15.8% fat, 1.25% cholesterol, 0.5% sodium cholate, and 7.5% casein for 3 weeks. During the feeding period of the lithogenic diet, mice were orally administrated with Hitrechol® preparation at once daily (QD, Group 4) or three times daily (TID, Group 5) and 50% PEG400 in H_2_O as vehicle control (Group 2) for 3 weeks. The dosing regimen for Group 1 to Group 5 mice was summarized in Fig. 1.

#### Sampling and sample treatment

At the end of 3-week treatment, mice from Groups 1 to 5 were sacrificed after overnight fasting followed by collecting their liver, gallbladder and bile. The sampling schedule for Group 1 to Group 5 mice was demonstrated in Fig. 1. Briefly, both body weight and liver weight of each mouse was recorded for calculation of Organ Index (Liver weight /Body weight). About 1 g of the liver from each mouse was minced and then homogenized using ultrasonic probe for 30 s on ice in 2 mL/g phosphate buffer saline for analyses of bile acid composition and antioxidative biomarkers including catalase, superoxide dismutase and reduced glutathione. The collected whole gallbladders were used to detect the bile acid composition, while the collected bile was used to determine the total cholesterol and phospholipid levels. For mice from Groups 1–4, blood was also collected via cardiac puncture followed by obtaining the serum after centrifugation at 8000 g for 3 min for preliminary analysis of antioxidative biomarkers including catalase, superoxide dismutase and reduced glutathione.

In addition, bile from Group 2 and Group 5 were immediately examined under a polarized microscope for cholesterol crystals morphology observation. Also, about 0.2 g of the liver from each mouse in Group 2 and Group 5 was immediately fixed in 4% pre-cooled paraformaldehyde for further histology treatment and observation. The median lobe of each mice livers from the above treatment groups were isolated and divided into two parts for Hematoxylin and Eosin (H&E) staining and Oil Red O staining, respectively. The first part of the median lobe was fixed in 4% paraformaldehyde at 4 °C until with further dehydration and embedding in paraffin wax. After being sectioned at 5 μm thickness and deparaffinized with xylene and graded alcohols, the mounted tissue sections were then stained with H&E for visualizing with 200 times magnification by a microscope (Eclipse Ti-E, Nikon, Japan) equipped with a digital video camera (DS-Qi2, Nikon, Japan) controlled by the NIS-Elements Imaging software program. Inflammation, necrosis, steatosis, cholestasis, and hemorrhage were major aspects during pathological observation [20]. The second part of the median lobe was stored at − 80 °C until the frozen section (6 μm) was obtained. Red Oil O staining was conducted to dye the lipids in liver section to red color. Two hundred times magnification was utilized for observation using the same microscope and imaging system mentioned for Red Oil O staining observation [20].

#### Evaluation of anti-lithogenic effect of Hitrechol®

##### Analyses of bile composition

In order to evaluate the effect of Hitrechol® on the composition of bile, levels of the total cholesterol, total phospholipids (0.2–0.5 μL) collected from Groups 1–5 were measured using Cholesterol/Cholesteryl Ester Quantitation Assay kit (ab65359) and Phospholipid Assay Kit (ab234050) according to protocols provided by the manufacturer (Abcam, USA) as described before [21, 22].

##### Analyses of bile acid composition in gallbladder and liver

To evaluate the effect of Hitrechol® on altering the bile acid composition, levels of the 12 bile acids in gallbladder and liver of mice from Groups 1–5 were measured and compared. Briefly, about 1 ml of 70% acetonitrile was added to the collected whole gallbladder organ followed by centrifugation at 13,000 g for 10 min before LC/MS/MS analyses of the bile acids levels. In addition, two volumes of acetonitrile were added to the prepared liver homogenate of mice from Groups 1–5 followed by centrifugation at 13,000 g for 10 min and vacuum drying of the supernatant. The obtained residue was then reconstituted in 70% acetonitrile followed by LC/MS/MS analyses of the bile acid levels in liver.

A previously reported LC/MS/MS method was adopted with modifications for simultaneous determination of 12 bile acids for the mentioned samples [23]. Briefly, the LC/MS/MS system consisted of an Agilent 6430 triple quadrupole mass spectrometer with an electrospray ionization source (ESI), Agilent 1290 pump and auto-sampler (Agilent Technologies Inc., USA). Chromatographic separation of the four analytes were achieved on a Welch Materials Ultimate HPLC XB-C18 (2.1 × 100 mm, 3 μm) analytical column. The mobile phase of water containing 7.5 mM ammonium formate adjusted with NaOH to pH = 7.0 (A) and methanol (B) were used with a gradient elution from 40% solvent B to 90% solvent B within 22 min. The flow rate was set at 0.3 ml/min. The injection volume of each sample was 20 μl. All bile acids were detected under the negative ionization mode with the following mass spectrometer source settings: ion spray voltage of − 4000 V; source temperature of 600 °C, collision gas pressure set at high; Q1/Q3 resolution set at high and interface heater turned on. The multiple reaction monitoring (MRM) transitions for each analyte as well as their respective optimum parameters, such as precursor ion, product ion, fragment voltage (Frag) and collision energy (CE) were listed in Table 1.

**Table 1:** MS/MS parameters for the detection of bile acids

Bile acids	Precursor Ion	Product Ion	Frag (V)	CE (V)	Polarity
Glycin-conjugated CA	464	74	100	70	Negative
Glycin-conjugated CDCA/UDCA/DCA	448	74	220	70	Negative
Taurin-conjugated CA	514	80	320	90	Negative
Taurin-conjugated CDCA/UDCA/DCA	498	80	125	80	Negative
Unconjugated CA	407	407	300	5	Negative
Unconjugated CDCA/UDCA/DCA	391	391	280	5	Negative

*CA* Cholic acid; *CDCA* Chenodeoxycholic acid; *UDCA* Ursodeoxycholic acid; *DCA* Deoxycholic acid

#### Evaluation of anti-oxidative and anti-inflammatory effects of Hitrechol®

To evaluate the anti-oxidative effect of Hitrechol®, catalase (CAT) activities, and superoxide dismutase (SOD) and reduced glutathione (GSH) levels were detected in collect serum and prepared liver homogenate from Groups 1 to 5 using Catalase Activity Assay Kit (ab83464), Superoxide Dismutase Activity Assay kit (ab65354) and GSH/GSSG Ratio Detection Assay kit (ab138881) according to the instructions of the manufacturer (Abcam, USA). Furthermore, to evaluate the anti-inflammatory effect of Hitrechol®, the concentration of TNF-alpha in serum were detected using ELISA kit (Abcam, USA).

#### Data analyses

All data reported in this study were expressed as mean value with standard deviation except for Organ Index, total cholesterol, total phospholipid and total bile acid levels, where mean value with standard error was adopted. The obtained levels of biomarkers and bile composition were compared among different treatment groups using one-way ANOVA tests followed by Tukey’s multiple comparisons with *p* < 0.05 considered as statistically significant.

## Results

### Effect of Hitrechol® on bile secretion flow rate in rats

**Fig. 2:**
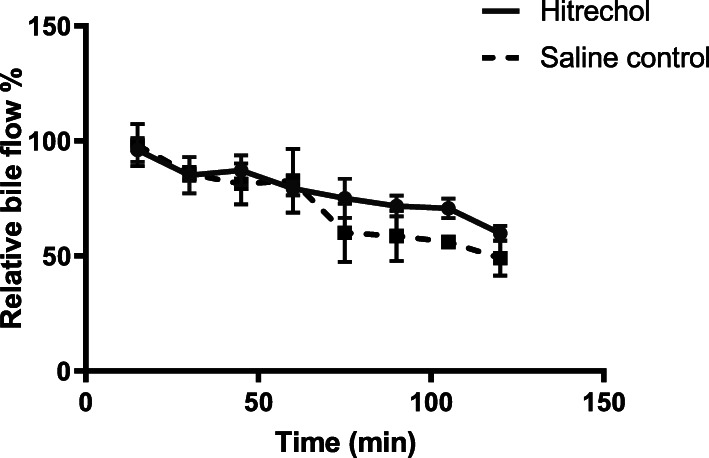
Comparison of Relative Bile Flow at different time intervals after oral administrations of Hitrechol® and saline to normal rats

As shown in Fig. 2, compared with the control group, slight elevation of bile flow in Hitrechol® treated group was observed at 105 min and 120 min post dosing. However, there is no statistically significant difference between the two groups after a multiple t-test conducted for each time point.

### Effect of Hitrechol® against the development of gallstone in normal and cholesterol gallstone model mice

#### Comparison of liver organ index from different treatment groups

**Fig. 3:**
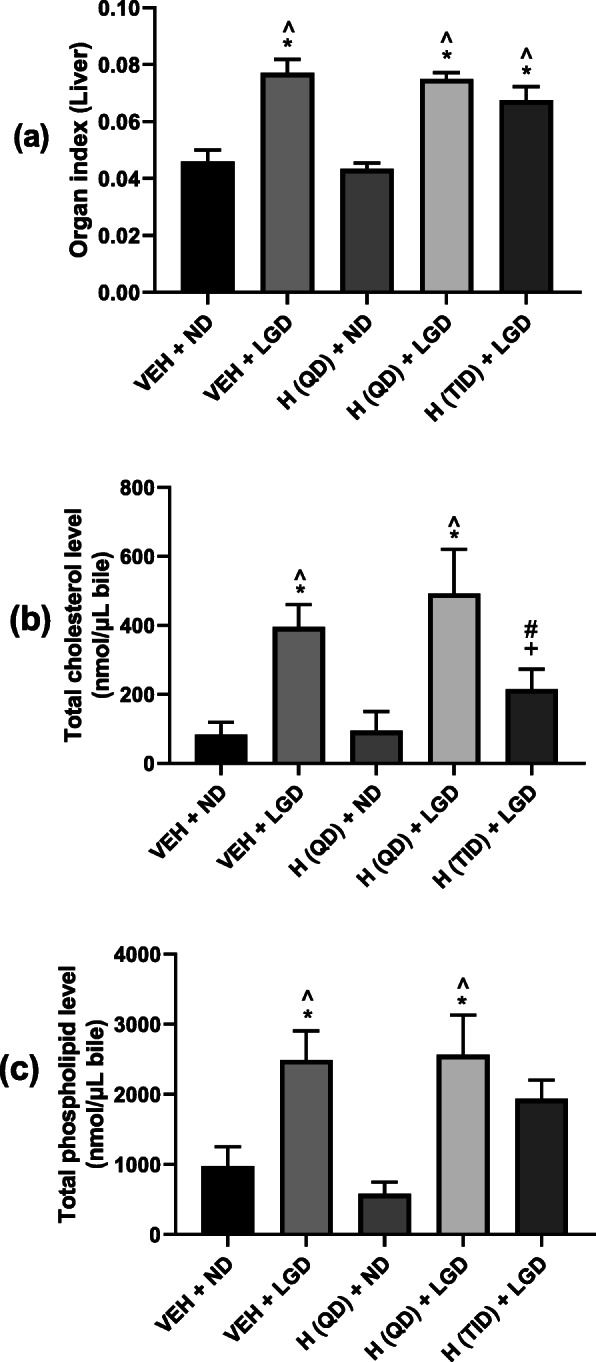
Effect of Hitrechol® on Organ Index (**a**), Total Cholesterol Levels (**b**) and Total Phospholipid Levels (**c**) of different treatment groups. *n* = 4 to 6 for each treatment group. Data presented in Mean ± SEM, *p < 0.05 compared to VEH + ND group, ^*p < 0.05* compared to H (QD) + ND group, *#p < 0.05* compared to VEH + LGD group, *+p < 0.05* compared to H(QD) + LGD group analyzed by one-way ANOVA followed by Tukey’s multiple comparisons test. VEH: Vehicle; ND: Normal diet; LGD: Lithogenic diet; H (QD): Hitrechol® once daily; H (TID): Hitrechol® three times daily. VEH + ND: Group 1; VEH + LGD: Group 2; H (QD) + ND: Group 3; H (QD) + LGD: Group 4; H (TID) + LGD: Group 5

As demonstrated in Fig. 3 (a), liver index of mice in all lithogenic diet groups were significantly increased compared to that of mice in normal diet groups. Among the three lithogenic groups (Groups 2, 4 & 5), it was found that Hitrechol® TID treated group (Group 5) showed a trend of decrease in liver index compared to the vehicle treatment group (Group 2), however there was no statistically significant difference.

#### Effect of Hitrechol® on Total cholesterol and phospholipid levels in bile

As shown in Fig. 3 (b), the total cholesterol levels in vehicle (Group 2) and Hitrechol® (QD) treated lithogenic diet groups (Group 4) were significantly elevated in comparison to that from normal diet groups (Groups 1 and 3). Although there was no significant difference in total cholesterol level between vehicle treated lithogenic diet group (Group 2) and Hitrechol® (QD) treated lithogenic diet group (Group 4), Hitrechol® TID treatment (Group 5) significantly decreased total cholesterol level in comparison to both vehicle (Group 2) and Hitrechol® (QD) (Group 4) treated lithogenic diet groups.

Figure 3 (c) demonstrated the comparison of total phospholipid among different treatment groups. It was noticed that the total phospholipid levels in vehicle and Hitrechol® (QD) treated lithogenic diet group (Group 4) were significantly elevated in comparison to that from normal diet groups (Groups 1 and 3). A trend of decrease in total phospholipid level was noticed in Hitrechol® TID (Group 5) in comparison to vehicle treated lithogenic diet group (Group 2) and Hitrechol® (QD) treated Lithogenic diet group (Group 4), however, there was no statistically significant difference.

#### Gallstone morphological and liver pathological observations after TID Hitrechol® treatment

**Fig. 4:**
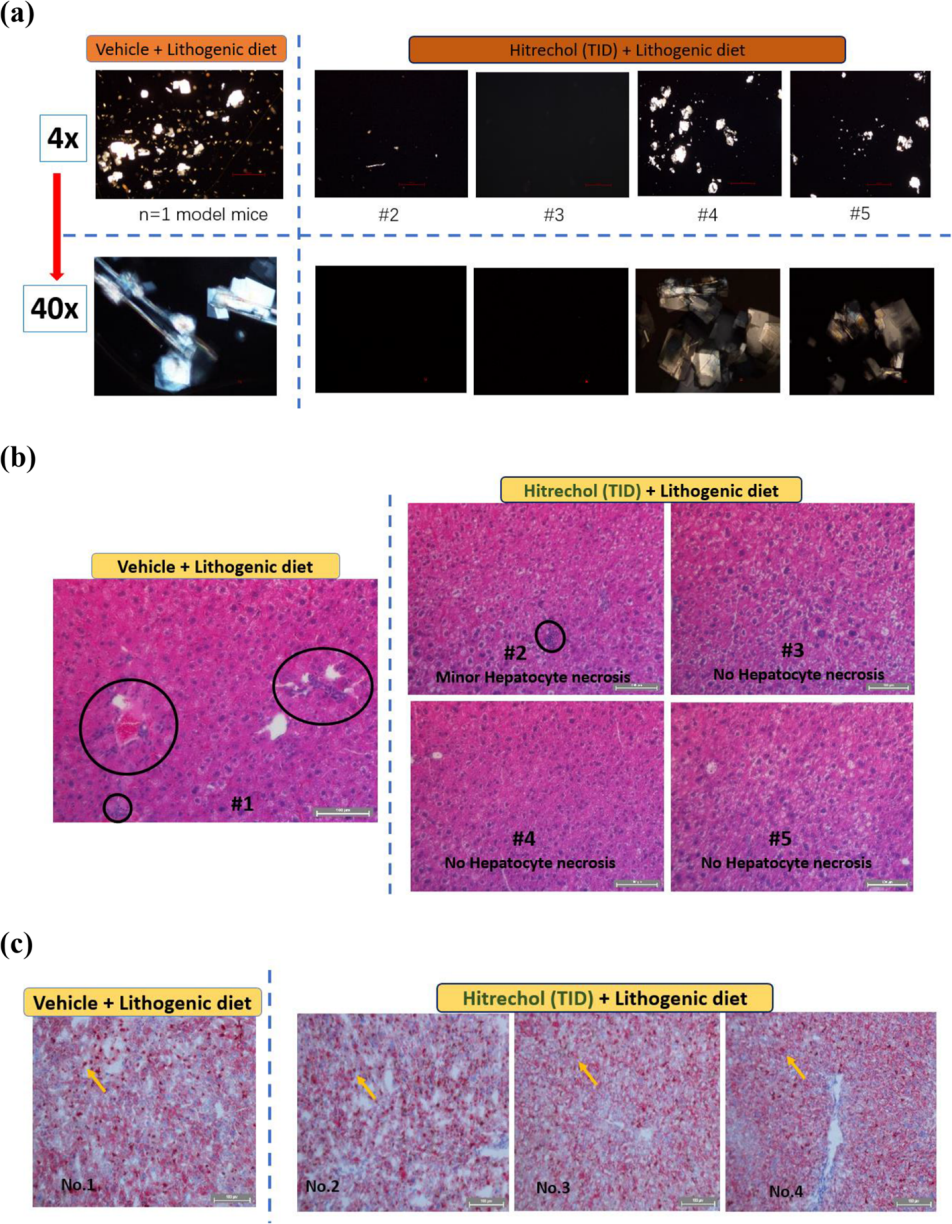
Polarizing light microscopy of gallstone (Cholesterol) crystals (**a**), Representative images of H&E-stained sections of livers (**b**) and Oil Red O staining of liver sections (**c**) from vehicle (Group 2) and Hitrechol® (TID) (Group 5) treated lithogenic diet groups. **Yellow Arrows** designates oil droplet. **Circles** indicate hepatocyte cell necrosis (scale bar indicates 100 μm)

Since the Hitrechol® TID treatment demonstrated significant improvements in antioxididative biomarkers and bile composition, we further observed their morphological and pathological changes. As shown Fig. 4 (a), more gallstone crystals were noticed in vehicle treated lithogenic diet mice than that of Hitrechol® TID treatment under 4 times magnification microscopy. Also, the morphology of crystals in vehicle treated lithogenic diet mice was found more rigid and larger under 40 times magnification microscopy. In addition, in Fig. 4 (b), despite observed severe hepatocyte necrosis in vehicle treated lithogenic diet group, minimal necrosis was found in TID Hitrechol® treated lithogenic diet group. Moreover, as demonstrated in Fig. 4 (c), oil droplets were in dark red color, prominent and widespread in vehicle treated lithogenic diet liver tissue. However, oil droplets in Hitrechol® TID treated lithogenic diet liver tissue were observed in lighter red color, smaller size compared with vehicle treated lithogenic diet one which indicated that Hitrechol® TID treatment could notably decrease cell necrosis chances and lipid aggregation in the liver.

#### Effect of Hitrechol® on bile acid compositions in gallbladder of lithogenic treated groups

**Fig. 5:**
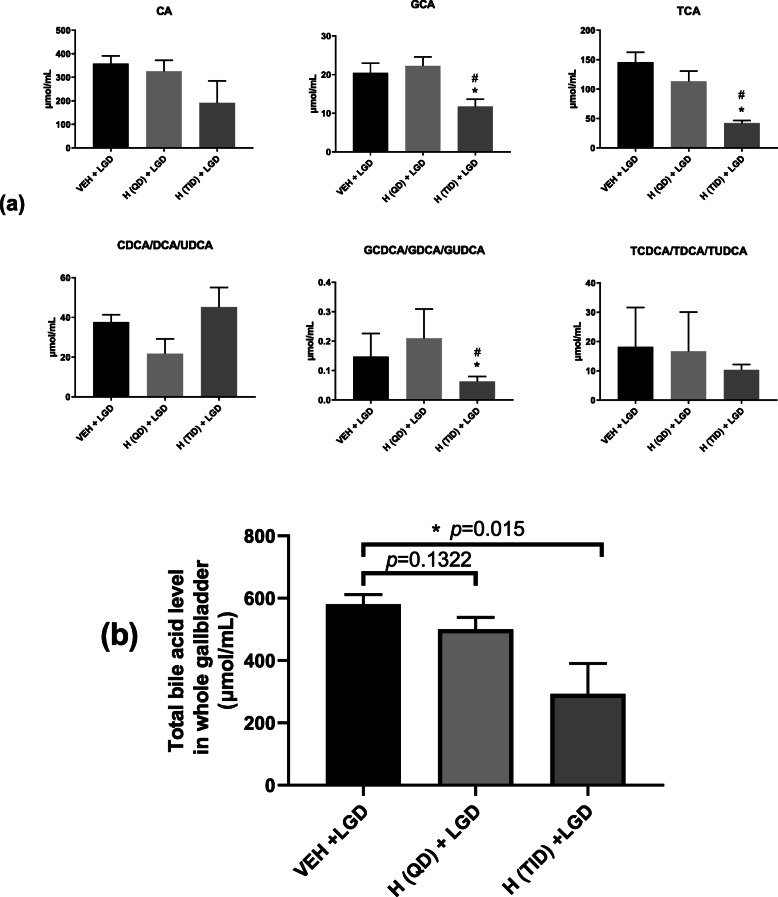
Comparison of detectable bile acid levels (**a**) and total bile acid levels (**b**) in the gallbladder of the three lithogenic diet treated groups. *n* = 4 to 6 for each treatment group. Data presented in Mean ± SEM, *p < 0.05 compared to VEH + LGD group analyzed by multiple t-test. VEH: Vehicle; LGD: Lithogenic diet; H (QD): Hitrechol® once daily; H (TID): Hitrechol® three times daily. VEH + LGD: Group 2; H (QD) + LGD: Group 4; H (TID) + LGD: Group 5

As shown in Fig. 5 (a), among the three lithogenic diet groups, significant decrease in the amount of GCA, TCA and GCDCA/GDCA/GUDCA in the whole gallbladder were found after TID but not QD Hitrechol® treatment. For the total bile acid level in the three lithogenic diet groups (Groups 2, 4 & 5), as demonstrated in Fig. 5 (b), significant reduction in total bile acid level was found in Hitrechol® (TID) group in comparison to that in vehicle treated group. Although Hitrechol® QD treatment group showed a trend of decrease compared to vehicle treated group, no statistical difference was found.

#### Anti-oxidative and anti-inflammatory effects of Hitrechol®

**Fig. 6:**
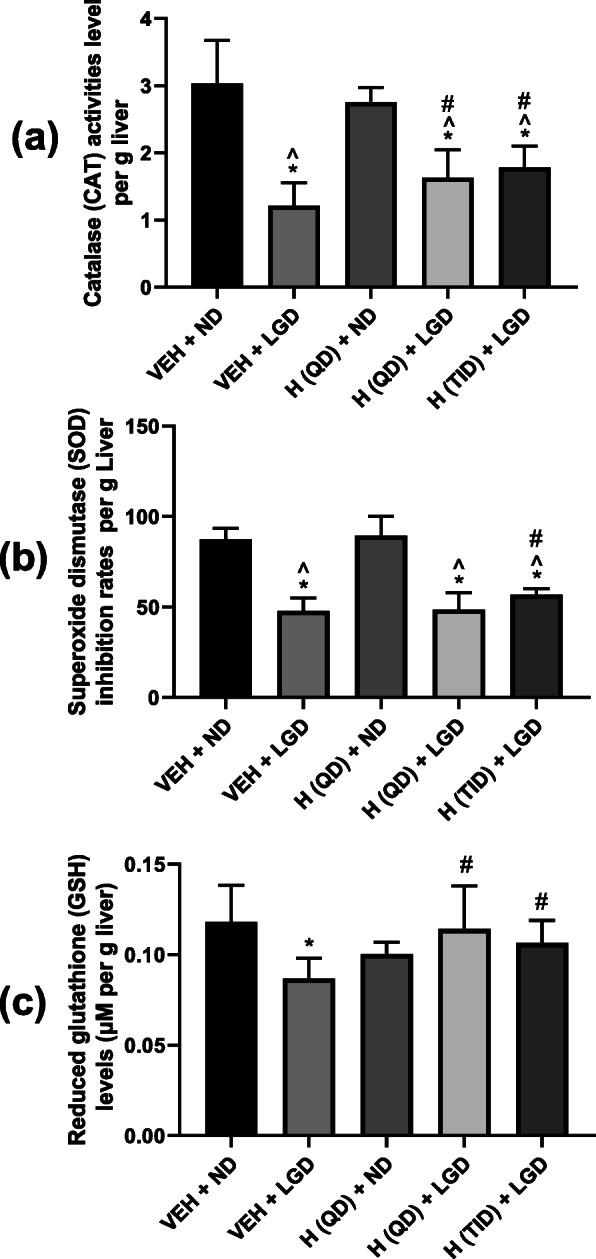
Comparisons of Catalase (CAT) activities (**a**), Superoxide dismutase (SOD) inhibition rates (**b**) and Reduced glutathione (GSH) levels (**c**) in liver from different treatment groups for evaluating the anti-oxidative effect of Hitrechol®. *n* = 4 to 6 for each treatment group. Data presented in Mean ± SD, *p < 0.05 compared to VEH + ND group, ^*p < 0.05* compared to H (QD) + ND group, *#p < 0.05* compared to VEH + LGD group analyzed by one-way ANOVA followed by Tukey’s multiple comparisons test. VEH: Vehicle; ND: Normal diet; LGD: Lithogenic diet; H (QD): Hitrechol® once daily; H (TID): Hitrechol® three times daily. VEH + ND: Group 1; VEH + LGD: Group 2; H (QD) + ND: Group 3; H (QD) + LGD: Group 4; H (TID) + LGD: Group 5

Since our preliminary evaluation found no significant difference in the serum level of CAT, SOD and GSH among the treatment groups of Groups 1, 2, 3 & 5, our subsequent comparison focused on their levels in the liver. As indicated in Fig. 6 (a), all the lithogenic diet groups (Groups 2, 4 & 5) had significantly decreased Catalase (CAT) activities level when compared with normal diet groups (Groups 1 and 3). In addition, it was also noticed that both QD and TID Hitrechol® (Groups 4 and 5) treatments significantly elevated the liver CAT activities level. As indicated in Fig. 6 (b), all the lithogenic diet groups (Groups 2, 4 & 5) exhibited significantly decrease in Superoxide dismutase (SOD) inhibition rates when compared with that from normal diet groups (Groups 1 and 3). It was also noticed that significant increase in SOD level in TID but not QD Hitrechol® treated group in comparison to vehicle treated lithogenic diet group. In Fig. 6 (c), both vehicle-treated lithogenic diet group (Group 2) and QD Hitrechol® treated normal diet group (Group 4) were found decrease in Reduced glutathione (GSH) levels compared to vehicle treated normal diet group (Group 1). In comparison to vehicle treated lithogenic group (Group 2), significant increase in Reduced GSH after Hitrechol® (QD) and Hitrechol® (TID) treatments, but no significant difference was found between QD and TID Hitrechol® treatment.

## Discussions

Gallstone formation is caused by the precipitation of bile components formed within the gallbladder with cholesterol precipitations as the most common one. Although Hitrechol® is used for the treatment of cholesterol gallstone disease since 1970s, the underlying mechanisms of actions of Hitrechol® for cholesterol gallstone treatment are still not clear. In this study, we investigated the effect of Hitrechol® on the bile flow in normal rats and compositions of bile alteration after Hitrechol® treatment in gallbladder in normal and cholesterol gallstone diseased mice. The morphology changes of gallstone and pathology changes in liver tissues cholesterol gallstone diseased mice provided more insights for the actions of Hitrechol® and specific antioxidative and anti-inflammatory biomarkers were measured to understand the hepatoprotective effect of Hitrechol®.

Based on the previous report on bile secretion increase by ursolic acid in normal rats [9], it was expected that Hitrechol® with ursolic acid as a major component might also be able to increase the bile secretion in liver via enhancing the bile flow. However, in the current experiment, despite a trend of slight elevation of bile flow for each time interval in Hitrechol® treated group observed at 105 min and 120 min postdosing, no statistical significance was found in comparison to that from the control group in normal rats. Such discrepancy may be due to the different treatment doses of ursolic acid. In the above-mentioned study [9], the lowest dose of ursolic acid was 7.5 mg/kg, whereas a much lower dose of ursolic acid (120 mg/kg Hitrecol® containing about 0.096 mg ursolic acid) was given to the rats in our current study.

Based on the indication of Hitrechol®, gallstone mice model was adopted for the current study for the evaluation of its impact on the gallstone formation. The successful development of the gallstone diseased mice model was verified by the gallstone formation shown in Fig. 4 (a) after mice were sacrificed in lithogenic diet treated groups. Obvious differences between mice treated with normal diet and treated with lithogenic diet were noticed in the biomarkers such as total cholesterol level and total phospholipid level [23]. In the normal diet groups, Hitrechol® treatment had minimal impact on the bile compositions such as the total cholesterol level, total phospholipid level and the total bile acid level with no significant changes in liver biomarkers including CAT, SOD and reduced-GSH levels, indicating the safe use of Hitrechol® with no severe side effects. Therefore, we focused on the subsequent comparisons on bile composition changes and biomarker changes in liver among the three lithogenic diseased groups. It was noted that the organ index for liver was all significantly increased in the three lithogenic groups, further suggesting the existence of liver injury. Moreover, in comparisons to the vehicle treated lithogenic group, the anti-lithogenic and liver protection effects of Hitrechol® both QD and TID treated groups could be significantly noticed in bile compositions and relevant biomarkers. For Hitrechol® QD treatment, it could potentially exhibit liver protection effect by increasing the CAT activity and reduced-GSH content in liver. Although no statistically significant decrease in total cholesterol and no significant increase in phospholipid levels after Hitrechol® QD treatment were noted, the gallstones were found in general to be smaller in size comparing to that in the vehicle treated group. After increasing the dosing frequency of Hitrechol® to TID, more obvious protective effect against the gallstone formation was noted. In addition, as demonstrated in Fig. 4 (b) and Fig. 4 (c) of liver histology samples, Hitrechol® TID treatment could notably decrease cell necrosis chances and lipid aggregation in liver caused by high fat and high cholesterol lithogenic diet, which may be contributed by oleanolic acid (70–350 μg/0.2 g capsule, 0.035–0.175% in Hitrechol®), a hepatoprotective drug for over 20 years in China [6, 24]. Besides the liver histology observations, we also noticed that the anti-oxidative effect in liver indicated by changes in CAT, SOD and Reduced GSH levels were further improved in Hitrechol® TID treatment group compared to Hitrechol® QD and vehicle treatment groups due to the liver protection effects of Hitrechol®. The anti-lithogenic effect of Hitrechol® could be reflected by the significant reduction of total cholesterol levels and slightly decreased phospholipid levels in the liver in Hitrechol® TID treatment group. As we mentioned previously [2], in normal physiological condition, bile salts, phospholipids, cholesterol, proteins, and bilirubin were all in stable equilibrium states. The decreased cholesterol level together with the increased phospholipid-to-bile acid ratio after Hitrechol® treatment is considered to lower the chances of cholesterol precipitation and subsequent gallstone formation. A previous study reported that higher ratio between phospholipid and bile acid could help to increase the solubility of cholesterol [25]. The mean value of phospholipid-to-bile acid ratio in vehicle treated groups was 4.35, which was increased to 4.91 after Hitrechol® QD treatment and significantly increased to 6.32 after Hitrechol® TID treatment. Such increase ratio between phospholipid-to-bile acid after Hitrechol® treatment could help to increase the solubility of cholesterol in bile and prevent the formation of cholesterol crystals. In addition, the decreased cholesterol level might also be contributed by linolenic acid (40–150 μg/0.2 g capsule, 0.02–0.075% in Hitrechol®), which was an essential fatty acid belonging to the omega-3 fatty acids group. It was reported that linoleic acid could lower the total cholesterol, low-density lipoprotein (LDL) cholesterol and high-density lipoprotein (HDL) cholesterol levels significantly [26, 27]. Normally, bile secretion was promoted to dissolve or lysis the gallstone when taking a high cholesterol diet. As expected, the total bile acid level in all the three lithogenic diet treated groups was noticed significantly increased compared to that of normal diet treated groups. In this study, after Hitrechol® TID treatment, the frequency of occurrence was significantly lowered with altered morphology of gallstone. Also, the levels of GCA, TCA and GCDCA/GDCA/GUDCA and total bile acid in gallbladder significantly decreased when compared to that from vehicle-treated group. It was worth noting that the UDCA (ursodeoxycholic acid) amount in bile acid composition showed a trend of increase after Hitrechol® TID treatment, which could be due to the presence of ursolic acid in Hitrechol®. The chemical structure of ursolic acid (40–150 μg/0.2 g capsule, 0.02–0.075% in Hitrechol®) is similar to that of ursodeoxycholic acid (UDCA), one of the endogenous bile acids that had been reported to be effective in dissolving gallstones via lysis of the cholesterol crystals [8].

## Conclusions

Hitrechol® dose-dependently demonstrated the anti-lithogenic effect and exhibited obvious liver protection effect through elevating the antioxidant biomarkers in cholesterol gallstone disease mice. Among the tested dosing regimens, Hitrechol® TID could effectively decrease the occurrence of gallstone formation and enhance the liver protection effect via altering the bile composition, elevating the antioxidative biomarkers and inhibiting IFN-gamma release.

## Supplementary Information

**Additional file 1: Fig. S1**. HPLC Chromatogram of the standard mixture of the major components in Hitrechol (a) and Hitrechol® capsules (b). **Table S1**: Certificate of Analysis of Hitrechol® (Batch No. 25003).

## Data Availability

All data generated during this study are included in this article. The fingerprint profile of the major components in Hitrechol® and its certificate of analyses are provided as supplementary information Fig. S1 and Table S1, respectively.

## Abbreviations

CA: Cholic acid
CDCA: Chenodeoxycholic acid
DCA: Deoxycholic acid
GSH: Reduced glutathione
H: Hitrechol®
LGD: Lithogenic diet
LC/MS/MS: Liquid chromatography with tandem mass spectrometry
N: Normal diet
QD: Once daily
SOD: Superoxide dismutase
TID: Three times daily
UDCA: Ursodeoxycholic acid

## References

[1] Lammert F, Gurusamy K, Ko CW, Miquel J, Mendez-Sanchez N (2016). Gallstones. Nat Rev Dis Primers.

[2] Maurer KJ, Carey MC, Fox JG (2009). Roles of infection, inflammation, and the immune system in cholesterol gallstone formation. Gastroenterology.

[3] Portincasa P, Moschetta A, Palasciano G (2006). Cholesterol gallstone disease. Lancet.

[4] Portincasa P, Ciaula AD, Bonfrate L, Wang DQ (2012). Therapy of gallstone disease: what it was, what it is, what it will be. World J Gastrointest Pharmacol Ther.

[5] Chen G, Wu S. Role of Baicalin and Liver X Receptor Alpha in the Formation of Cholesterol Gallstones in Mice. Gastroenterol Res Pract. 2020;1343969. 10.1155/2020/1343969. 10.1155/2020/1343969 PMC7191361 32382260

[6] Chou ST, Lin TH, Peng HY, Chao WW. Phytochemical profile of hot water extract of Glechoma hederacea and its antioxidant, and anti-inflammatory activities. Life Sci. 2019;231:116519. 10.1016/j.lfs.2019.05.075. 10.1016/j.lfs.2019.05.075 31152813

[7] Kitani K, Kanai S, Ivy G, Carrillo MC (1999). Pharmacological modifications of endogenous antioxidant enzymes with special reference to the effects of deprenyl: a possible antioxidant strategy. Mech Ageing Dev.

[8] Podda M, Zuin M, Battezzati PM, Ghezzi C, Fazio DC, Dioguardi ML (1989). Efficacy and safety of a combination of chenodeoxycholic acid and ursodeoxycholic acid for gallstone dissolution: a comparison with ursodeoxycholic acid alone. Gastroenterology..

[9] Xiong X, Chen W, Cui J, Yi S, Zhang Z, Li K (2003). Effects of ursolic acid on liver-protection and bile secretion. Zhong Yao Cai.

[10] Chen P, Li J, Fan X, Zeng H, Deng R, Li D, Huang M, Bi H (2015). Oleanolic acid attenuates obstructive cholestasis in bile duct-ligated mice, possibly via activation of NRF2-MRPs and FXR antagonism. Eur J Pharmacol.

[11] Hu G, Yuan X, Zhang S, Wang R, Yang M, Wu C, Wu Z, Ke X (2015). Research on choleretic effect of menthol, menthone, pluegone, isomenthone, and limonene in DanShu capsule. Int Immunopharmacol.

[12] An HJ, Jeong HJ, Um JY, Kim HM, Hong SH (2006). Glechoma hederacea inhibits inflammatory mediator release in IFN-gamma and LPS-stimulated mouse peritoneal macrophages. J Ethnopharmacol.

[13] Bergendorff O, Franzén C, Jeppsson AB, Sterner O, Waldeck B (1995). Screening of some European medicinal plants for spasmolytic activity on isolated Guinea-pig trachea. Int J Pharmacol.

[14] Wang YY, Lin SY, Chen WY, Liao SL, Wu CC, Pan PH, Chou ST, Chen CJ (2017). Glechoma hederacea extracts attenuate cholestatic liver injury in a bile duct-ligated rat model. J Ethnopharmacol.

[15] Casey HJ. Efficacy study on gallstones remedy, vegetable extract II, final report of the study 1976; ref. no.11/7606, Pharmazeutische Fabrik Evers & Co GmbH.

[16] Milovanovic M, Zivkovic D, Vucelic-Radovic B (2010). Antioxidant effects of Glechoma hederacea as a food additive. Nat Prod Commun.

[17] Kumarasamy Y, Cox PJ, Jaspars M, Nahar L, Sarker SD (2002). Biological activity of Glechoma hederacea. Fitoterapia.

[18] Ohigashi H, Takamura H, Koshimizu K, Tokuda H, Ito Y (1986). Search for possible antitumor promoters by inhibition of 12-O-tetradecanoylphorbol-13-acetate-induced Epstein-Barr virus activation; Ursolic acid and oleanolic acid from an anti-inflammatory Chinese medicinal plant, *Glechoma hederacea*e L. Cancer Lett.

[19] Fuchs-Algrim J, Lorenz H. Efficacy and tolerability of Hitrechol® in the dissolution of cholesterol gallstones and the treatment of accompanying complaints. 2003 Integrated final report of study IBP 312001/93.

[20] Kleiner DE, Chalasani NP, Lee WM, Fontana RJ, Bonkovsky HL, Watkins PB, Hayashi PH, Davern TJ, Navarro V, Reddy R, Talwalkar JA, Stolz A, Gu J, Barnhart H, Hoofnagle JH, for the Drug‐Induced Liver Injury Network (DILIN) (2014). Hepatic histological findings in suspected drug-induced liver injury: systematic evaluation and clinical associations. J Hepatol.

[21] Cheng S, Zou M, Liu Q, Kuang J, Shen J, Pu J (2017). Activation of constitutive androstane receptor prevents cholesterol gallstone formation. Am J Pathol.

[22] Carey MC (1978). Critical tables for calculating the cholesterol saturation of native bile. J Lipid Res.

[23] Huang J, Sai PR, Bathena IL, Csanaky YA (2011). Simultaneous characterization of bile acids and their sulfate metabolites in mouse liver, plasma, bile, and urine using LC–MS/MS. J Pharm Biomed Anal.

[24] Rassias G (1991). Kestin M Nestel PJ linoleic acid lowers LDL cholesterol without a proportionate displacement of saturated fatty acid. Eur J Clin Nutr.

[25] Moschetta A, van Berge-Henegouwen GP, Portincasa P, van Erpecum KJ GP (2001). Cholesterol crystallization in model biles: effects of bile salt and phospholipid species composition. J. Lipid Res.

[26] Zhao H, Zhou M, Duan L, Wang W, Zhang J, Wang D, Liang X (2013). Efficient synthesis and anti-fungal activity of oleanolic acid oxime esters. Molecules.

[27] Ayeleso TB, Matumba MG, Mukwevho E (2017). Oleanolic acid and its derivatives: biological activities and therapeutic potential in chronic diseases. Molecules..

[28] Percie du SN, Hurst V, Ahluwalia A, Alam S, Avey MT, Baker M, et al. The ARRIVE guidelines 2.0: updated guidelines for reporting animal research. PLoS Biol. 2020; 18(7): e3000410. 10.1371/journal.pbio.3000410 PMC7360023 32663219

