# Supplementary material for: Protective effect of Glechoma hederacea extract against gallstone formation in rodent models
Source: BMC Complement Med Ther. 2021 Jul 14;21:199. doi: 10.1186/s12906-021-03368-1 (PMC8278774; doi:10.1186/s12906-021-03368-1)
Supplement: Supplementary file 1 — Additional file 1: Fig. S1. HPLC Chromatogram of the standard mixture of the major components in Hitrechol (a) and Hitrechol® capsules (b). Table S1: Certificate of Analysis of Hitrechol® (Batch No. 25003). [file 12906_2021_3368_MOESM1_ESM.docx]

**(a)**


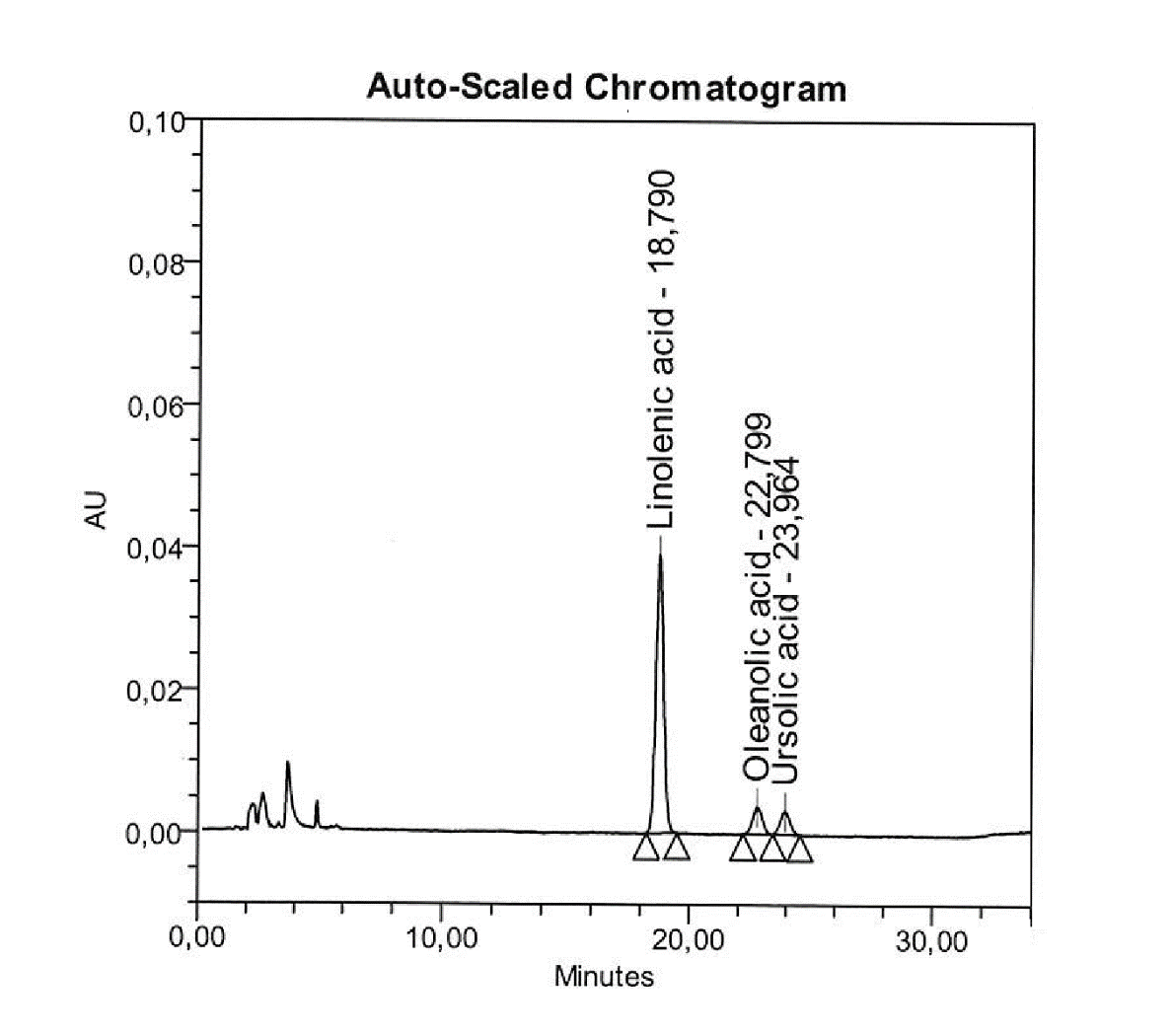


**(b)**


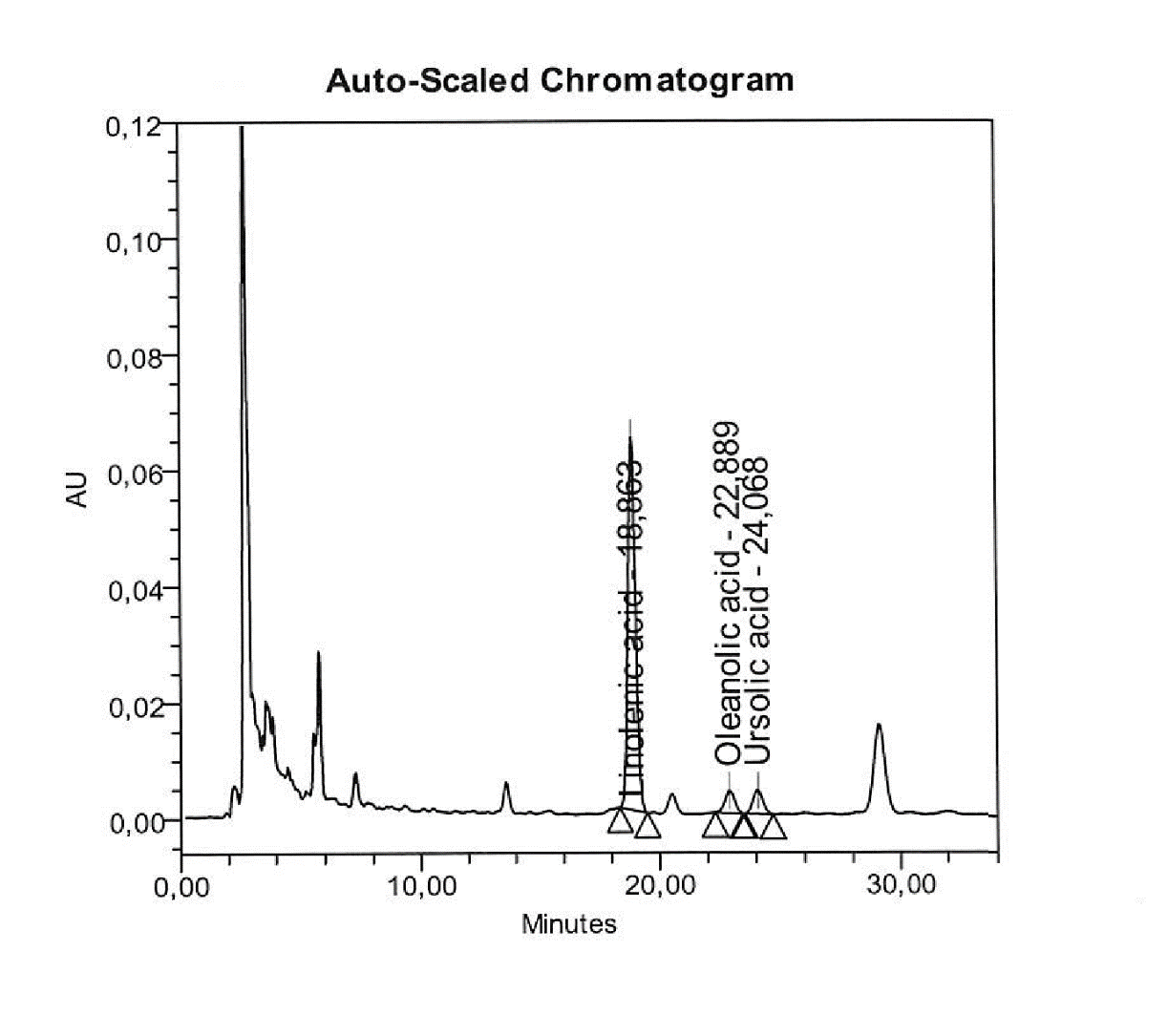


**Figure S1. HPLC Chromatogram of the standard mixture of the major components in Hitrechol (a) and Hitrechol^®️^ capsules (b).**

**Table S1: Certificate of Analysis of Hitrechol^®️^ (Batch No. 25003)**

| **Parameters** | **Specification** | **Results of Batch No. 25003** |
| --- | --- | --- |
| **Characteristics** | | |
| Description | Soft capsules with filling of yellow-green oily material, odour slightly fragrant | Complies |
| Filling variation | 200 mg (not more than 2 out of 20: ±10%,  none out of 20: ± 20%) |  |
| Disintegration time | Complies with the requirement for capsules |  |
| **Identity** | | |
| TLC fingerprint of active ingredient | TLC fingerprint has to comply | Complies |
| **Purity** | | |
| Appearance of filling mass | Yellow-green, oily | Complies |
| Microbial limit test | Bacteria count: not more than 1000 cfu per g  Fungi and yeasts count: not more than 100 cfu per g  *E. coli*: absence in 1 g  *Salmonella*: absence in 10 g | Complies |
| **Assay** | | |
| Sum of Ursolic acid and Oleanolic acid | 40 – 150 µg per capsule | Complies |
| Linolenic acid | 70 – 350 µg per capsule | Complies |
